# Longitudinal analysis of lung microbiome, immune response, and metabolism in ventilator-associated pneumonia: a cohort study

**DOI:** 10.1186/s13054-025-05498-1

**Published:** 2025-07-03

**Authors:** Ingrid G. Bustos, Cristian C. Serrano-Mayorga, José L. Guerrero, Jennifer M. Baker, Christopher Brown, Nicole Falkowski, Piyush Ranjan, Alejandro Acosta-Gonzalez, Lina M. Mendez, Acenet Garcia-Cordoba, Adriana Echeverry-Gutierrez, Denis A. Bojaca, Marcela Chisica-Mahecha, Nicol Guarin-Tequia, Liliana Romero-Romero, Norberto Gonzalez-Juarbe, Alejandro Rodriguez, Mónica P. Cala, Ignacio Martin-Loeches, Sanjay H. Chotirmall, Robert P. Dickson, Luis F. Reyes

**Affiliations:** 1 Unisabana Center for Translational Science, School of Medicine, Universidad de La Sabana, Chia, Colombia; 2 Engineering School, Universidad de la Sabana, Chia, Colombia; 3 Clinica Universidad de La Sabana, Chia, Colombia; 4 Met Core-Metabolomics Core Facility. Vice-Presidency of Research and Knowledge Creation, Universidad de Los Andes, Bogota, Colombia; 5 Division of Pulmonary and Critical Care Medicine, Department of Internal Medicine, University of Michigan Health System, Ann Arbor, MI 48109 USA; 6 Department of Microbiology and Immunology, University of Michigan Medical School, Ann Arbor, MI 48109 USA; 7 Weil Institute for Critical Care Research & Innovation, Ann Arbor, MI USA; 8 Advanced Research Computing (ARC)Information and Technology Services (ITS), University of Michigan, Ann Arbor, MI USA; 9 Department of Cell Biology and Molecular Genetics, University of Maryland, College park, MD USA; 10 Critical Care DepartmentURV/IISPV/CIBERES, Hospital Universitario Joan XXIII, Tarragona, Spain; 11 Multidisciplinary Intensive Care Research Organization (MICRO), St James Hospital, Dublin, Dublin Ireland; 12 Lee Kong Chian School of Medicine, Nanyang Technological University, Singapore, Singapore; 13 Department of Respiratory and Critical Care Medicine, Tan Tock Seng Hospital, Singapore, Singapore; 14 ISARIC, Pandemic Sciences Institute, University of Oxford, Oxford, UK

**Keywords:** Microbiota, Host microbial interaction, Metabolomics, Ventilator-Associated pneumonia (VAP)

## Abstract

**Rationale:**

Ventilator-associated pneumonia (VAP) is a common complication in patients under invasive mechanical ventilation (IMV), yet its underlying mechanisms remain poorly understood. This study investigated the lung microbiome, inflammatory response, and metabolism in patients undergoing IMV to identify factors that may predispose them to developing VAP.

**Objectives:**

To investigate how changes in lung microbiome composition, inflammatory response, and metabolic profiles may predispose patients undergoing IMV to develop VAP.

**Methods:**

Patients requiring IMV for at least 48 h due to non-infectious respiratory failure were enrolled. Bronchoalveolar lavage (BAL) samples were collected at baseline, upon VAP diagnosis, or after 72 h for non-VAP cases. DNA sequencing, cytokine quantification, and metabolomic analyses were performed.

**Results:**

Of the 80 patients, 41 (51%) developed VAP. No significant differences in alpha or beta diversity of the lung microbiome were observed between groups. However, both groups showed changes in microbiome composition over time, suggesting an impact of IMV. Tumour necrosis factor-alpha (TNF-α) lung levels were significantly higher in VAP patients, while lung interleukin-1 beta (IL-1β) increased in all patients. Metabolomic analysis revealed shifts in pentose phosphate and citric acid cycle pathways, indicating a transition to anaerobic metabolism in the lungs of VAP patients.

**Conclusions:**

Mechanical ventilation was associated with temporal changes in lung microbiome composition independent of VAP development. VAP cases exhibited higher TNF-α levels and metabolic profiles indicative of anaerobic adaptation, while IL-1β elevations were primarily linked to mechanical ventilation rather than infection.

**Supplementary Information:**

The online version contains supplementary material available at 10.1186/s13054-025-05498-1.

## Introduction

Ventilator-associated pneumonia (VAP) is a lung infection that develops after ≥ 48 h of invasive mechanical ventilation (IMV). It is primarily bacterial but can also be viral or fungal [1, 2]. VAP results from the micro-aspiration of oropharyngeal or gastric contents, disruption of mucociliary clearance, and pathogen translocation, which are exacerbated by immune impairment and biofilm formation on airway devices [3, 4]. VAP is the most common Intensive Care Unit (ICU)-acquired infection, affecting up to 40% of IMV patients [1, 5]. Its incidence varies by region, with 18 cases per 1,000 ventilator days in Europe and low-middle-income countries, compared to 1–2.5 cases per 1,000 in the USA [1, 6]. Mortality rates range from 18 to 50%, depending on patient factors and healthcare settings [6]. Additionally, VAP imposes a significant economic burden, with an estimated cost of $40,144 per case [7].

Recent studies have highlighted the interplay between the lung microbiome and the immune system, revealing a symbiotic relationship essential for pulmonary homeostasis and immune responses against pathogens [8]. However, identifying etiological pathogens in VAP remains challenging, and its underlying mechanisms remain unclear [9]. Advances in 16 S rRNA gene sequencing have enhanced our understanding of microbial communities in healthy and VAP individuals [10, 11], with alpha diversity (within-sample richness and evenness richness is the number of different taxa (e.g., species or operational taxonomic units) present in a community, while evenness describes how evenly the individual organisms are distributed among the different taxa and beta diversity (between-sample compositional differences; it reflects the extent of change or turnover in species composition from one community to another) providing insights into lung microbiome composition [12, 13]. Reduced alpha diversity within 48 h post-intubation has been associated with prolonged ventilation, but not with ventilator-associated pneumonia (VAP) or antibiotic use [14, 15]. While alterations in alpha and beta diversity have been proposed as indirect indicators of microbial dysbiosis in the lung [16, 17], they are not sufficient to reliably diagnose lower respiratory tract infections. Dysbiosis—defined as a disruption of the microbial community structure favouring potential pathogens—can modulate local immune responses, establishing [15, 18, 19]. This interaction may occur via bacterial metabolites that influence cytokine expression and host immunity [19, 20]. However, the causal mechanisms linking microbiome alterations to VAP remain unclear and warrant further investigation.

The interaction between the lung microbiome, immune cells, cytokines, chemokines, and metabolites is crucial for lung homeostasis and immune regulation [8, 19, 20]. In ICU patients, mechanical ventilation and antibiotics can disrupt the microbial balance, weakening host defences and increasing the risk of infection. Under conditions of dysbiosis, commensal microbes may acquire pathogenic traits and act as pathobionts, contributing to infection [21]. These observations underscore the dynamic nature of the pulmonary microbiome and support viewing it as a fluid continuum rather than a static entity, particularly in the ICU setting. This study aimed to analyse longitudinal changes in the lung microbiome, immune response, and metabolism in mechanically ventilated patients. We hypothesised that the early course of invasive mechanical ventilation contributes to disruptions in these factors, facilitating VAP pathogenesis. Understanding their individual roles can provide valuable insights into disease progression.

## Materials and methods

### Study protocol and population

**Fig. 1:**
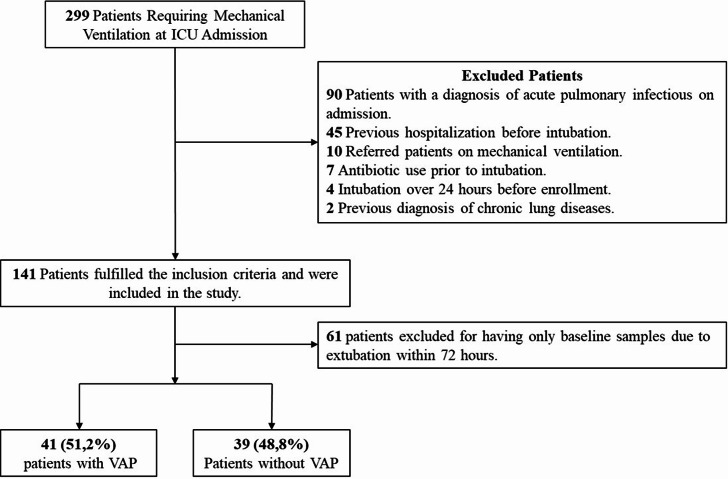
Flowchart of bronchoalveolar lavage (BAL) collected from the intensive care unit

This prospective, monocentric observational cohort study was conducted at Clinica Universidad de la Sabana, Chia, Colombia, from January 2020 to July 2022). The study adhered to institutional protocols and ethical guidelines, with approval obtained from the Institutional Review Board (IRB) (IRB 20190903, MED-468). Adult ICU patients (> 18 years) with acute non-infectious respiratory failure—defined as the absence of clinical or radiological evidence of pulmonary infection—and an anticipated need for ≥ 48 h of IMV were prospectively screened. Based on clinical judgment at the time of intubation, bronchoalveolar lavage (BAL) was performed within the first 12 h. Patients who remained intubated for 48 h or longer and had available follow-up BAL samples were included in the final analysis. Those extubated before 72 h were excluded due to the lack of longitudinal sampling (Fig. 1).

Exclusion criteria included pregnancy, breastfeeding, prior IMV > 24 h, recent antibiotic exposure (≤ 48 h), clinical or radiological evidence of pulmonary infection at baseline, chronic lung disease, or COVID-19-related respiratory failure. All patients were consecutively enrolled during the study period. Written informed consent was obtained from the patients’ legally authorised representatives, as all participants were critically ill and unable to provide consent. Clinical data were anonymised and managed using the REDCap system.

### Diagnostic criteria for VAP and sample collection

VAP was diagnosed using the Infectious Diseases Society of America (IDSA) and American Thoracic Society (ATS) guidelines [5], requiring radiographic infiltrate deterioration and two or more clinical criteria: fever (> 38 °C), purulent tracheal discharge, or leukocyte count changes (< 4,000/µL or > 10,000/µL). Microbiological confirmation of VAP in this cohort followed routine clinical practice. It was based on IDSA/ATS guidelines, requiring ≥ 10⁶ CFU/mL in endotracheal aspirates (ETA) or ≥ 10⁴ CFU/mL in BAL fluid, depending on the sample obtained by the treating clinical team. Importantly, all biological samples used for microbiome, cytokine, and metabolomic analyses in this study were exclusively collected from BAL fluid [5]. 

BAL and blood samples were collected within 12 h of intubation, following institutional protocols. BAL samples were obtained using a HALYARD^®^ bronchoscopy sampling catheter (reference REF142, Minerva Medical), and sample collection was performed using an Argyle^®^ 40 mL specimen trap (reference 8884724500). In all patients, 30 mL of sterile saline was instilled into the right middle lobe, using 20 mL and 10 mL aliquots depending on clinical tolerance. The average volume recovered ranges from 60 to 80% of the total instilled volume.

For patients who developed VAP, follow-up BAL samples were obtained at either 72 h or day five post-intubation, depending on the duration of intubation and the timing of the VAP diagnosis. The 72-hour time point was selected because most patients were not intubated beyond five days, and it aligns with the clinical definition of VAP, which requires pneumonia onset more than 48 h after intubation. All BAL samples were aliquoted immediately after collection and stored at − 80 °C within one hour, without prior centrifugation. This approach was used to avoid repeated freeze–thaw cycles and thereby minimise the risk of cell lysis. Blood samples were centrifuged to separate the serum, which was then stored at − 80 °C for subsequent analysis.

### DNA extraction, 16 S rRNA gene PCR amplification and sequencing

DNA was extracted from 500 µL of BAL fluid using the DNeasy^®^ Blood & Tissue Kit (QIAGEN) and quantified via NanoDrop™ One spectrophotometer. The V4 region of the 16 S rRNA gene was amplified using AccuPrime High-Fidelity Taq and sequenced on the Illumina MiSeq platform (2 × 250 bp) with minor protocol modifications. Bacterial DNA burden was assessed by QX200 Droplet Digital Polymerase Chain Reaction (ddPCR) (Bio-Rad). Reads were processed using Mothur v.1.48, following the MiSeq SOP pipeline [22]. OTUs were clustered at 97% similarity, and taxonomic assignments were made using the SILVA reference database (v138.1).

### Inflammatory profiling in the lower respiratory tract

Interleukin (IL)−1β, IL-6, and Tumour Necrosis Factor-alpha (TNF-α) levels in BAL fluid were measured to assess lower respiratory tract inflammation. To enhance accuracy, 100 µL BAL was mixed with 100 µL sputolysin. The Milliplex assay (Millipore Corp.) was performed according to the manufacturer’s guidelines using the High Sensitivity Human Cytokine kit (HCYTA-60 K, Millipore Corp.).

### Metabolomic profiles in the lower respiratory tract

BAL samples were centrifuged, and metabolites were extracted using a methanol-chloroform mixture. Plasma samples were analysed using gas chromatography time-of-flight mass spectrometry (GC-TOF-MS) with an Agilent quadrupole time-of-flight (QTOF) 7250 system. Data processing involves deconvolution, alignment, integration, and normalisation. Metabolites were identified using a metabolomics library, with experiments conducted at MetCore, Universidad de Los Andes, Bogotá, following published protocols [23, 24].

### Statistical analysis

Sequencing data were processed using Mothur v.1.42.3 (Schloss SOP), with operational taxonomic units (OTUs) classified at 97% identity via the Ribosomal Database Project (RDP) Classifier [22]. Principal coordinates analysis (PCoA) in R v.4.3.1 analysed OTUs > 0.5%, while group heterogeneity was assessed using chi-square, Fisher’s exact, T-tests, or Mann-Whitney U tests. Microbial diversity was assessed using the vegan package in R. Alpha diversity was evaluated with Shannon, Simpson, and Chao1 indices; beta diversity was calculated with Bray–Curtis dissimilarity and tested for group differences using permutational multivariate analysis of variance (PERMANOVA). Multivariate analyses were performed using orthogonal partial least squares discriminant analysis (OPLS-DA) in SIMCA 16.0 and MATLAB R2019b, with false discovery rate (FDR) correction. Cross-validation and permutation tests confirmed model reliability.

## Results

Among the 299 mechanically ventilated patients screened, 141 were enrolled in the study. However, 61 were excluded because they were extubated within 72 h and thus only had baseline samples available, representing a comparatively healthier subgroup. The final cohort included 80 patients, 41 (51%) were diagnosed with VAP (Fig. 1). Demographic and clinical characteristics are presented in Table S1 and Table S2, and Figure S1 shows ICU admission causes, with traumatic brain injury and neurological conditions being the most frequent. The study population was predominantly male, comprising 68.8% (55 of 80), with a median age of 51.5 years (interquartile range: 34.0–69.2). The most common comorbidities were arterial hypertension (27 of 80 patients, 33.8%), diabetes mellitus (7 of 80, 8.8%), and a history of smoking (6 of 80, 7.5%). No patients in either group met criteria for immunosuppression due to underlying disease or use of immunosuppressive therapy. Additionally, 73 of 80 patients (91.2%) received antibiotic therapy either continuously or intermittently during their ICU stay. In most cases, antibiotics were administered prophylactically—often as a single dose—or as part of extended regimens in patients with polytrauma, open wounds, or those undergoing multiple surgical interventions. The cohort consisted of critically ill patients requiring prolonged mechanical ventilation and frequent antibiotic exposure, providing a relevant clinical context for the observed microbial and host response patterns.

In the comparative analysis of patients with VAP versus those without VAP (No-VAP), some differences were observed in clinical and demographic variables. Patients with VAP had a significantly longer ICU stay (15.0 vs. 10.0 days; *p* = 0.01) and hospitalisation length of stay (29.0 vs. 15.0 days; *p* < 0.01) compared to No-VAP patients. The duration of IMV was also significantly prolonged in the VAP group (10.0 vs. 6.0 days; *p* = 0.01). Physiological variables, such as heart and respiratory rates, were comparable within the first 12 h of intubation between groups. Glasgow Coma Scale scores were significantly higher in No-VAP patients (*p* = 0.04). Notably, baseline PaO2 did not differ significantly between groups (*p* = 0.07). Further details, including interquartile ranges and additional variables, are in Table S1.

### Taxonomic distribution and quality control of the 16 S rRNA sequence

A total of 20,808,915 sequence reads and 18,475 OTUs were obtained, averaging 130,873 reads per BAL sample. After filtering out OTUs with relative abundances below 0.1%, 2,096 OTUs remained, primarily assigned at the genus level. Rarefaction curves confirmed adequate sequencing depth (Fig. S2), with no significant differences between groups (*p* > 0.05). No-template control (NTC) samples exhibited lower diversity than BAL samples (*p* < 0.01). Absolute abundance analysis confirmed significantly higher bacterial DNA in BAL than in NTC samples, validating BAL as a suitable specimen for sequencing and ensuring reliable downstream analyses (Fig. S3).

### Alpha diversity of lower respiratory tract microbiota

**Fig. 2:**
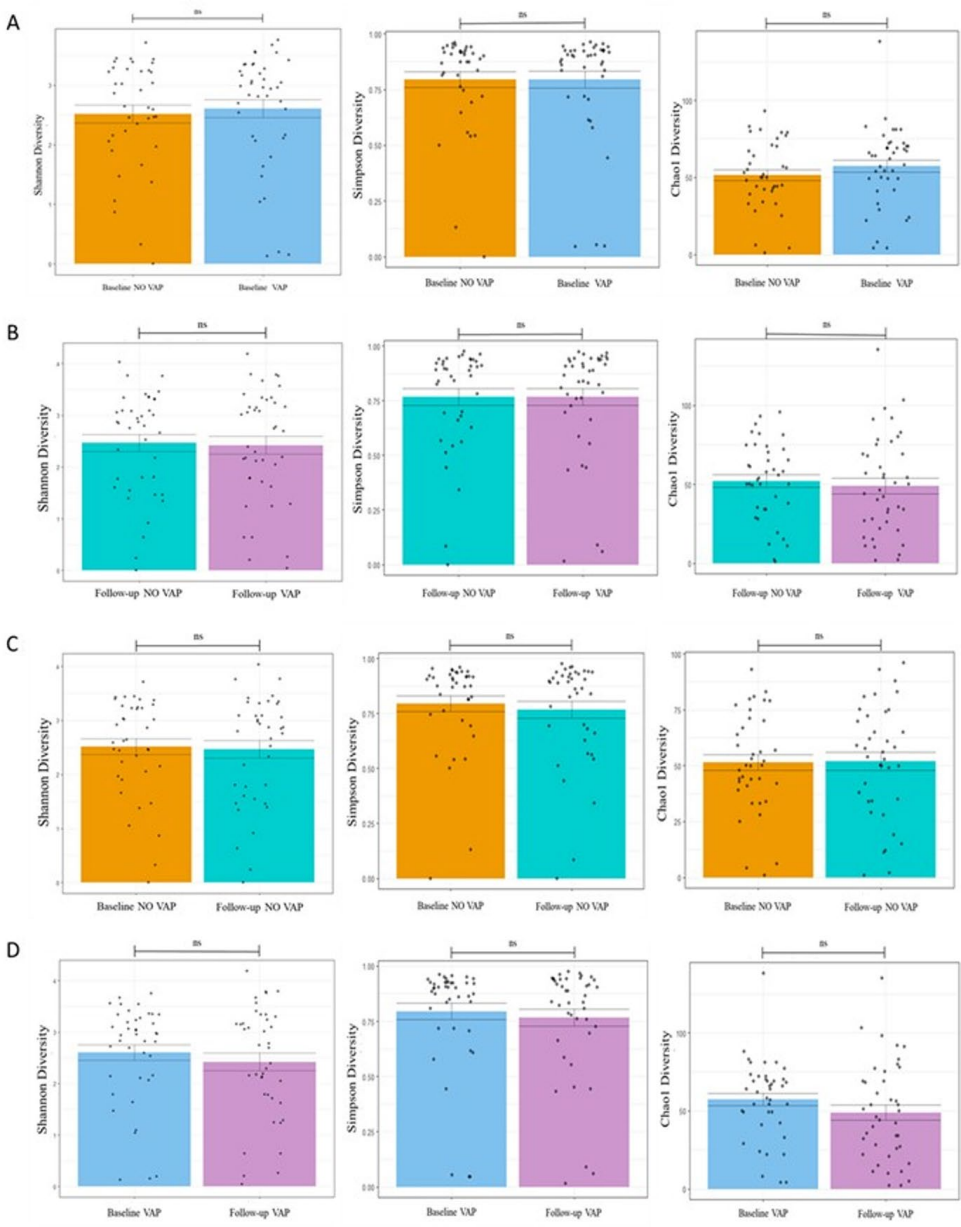
Alpha Diversity Comparisons. Plots illustrating alpha diversity indices (Shannon, Simpson, Chao1) in baseline and follow-up samples from VAP and non-VAP patients. (**A**) Baseline samples show no significant difference in alpha diversity between VAP and non-VAP groups. (**B**) Follow-up samples also show no significant difference. (**C**) Non-VAP patients exhibit no significant changes in alpha diversity between baseline and follow-up samples. (**D**) VAP patients similarly show no significant differences between baseline and follow-up samples. Asterisks denote the level of significance observed: * = *p* ≤ 0.05; ** = *p* ≤ 0.01; *** = *p* ≤ 0.001; ns = ≥ 0.05

Alpha diversity was assessed using Shannon, Simpson, and Chao1 indices in baseline and follow-up BAL samples. No significant differences were observed between patients who developed VAP and those who did not (*p* > 0.05 for all indices; Fig. 2A–B). Similarly, within each group, alpha diversity remained stable over time, with no significant changes between baseline and follow-up samples (*p* > 0.05 for all comparisons; Fig. 2C–D). No significant differences in microbial richness or evenness were observed across groups, regardless of VAP status or duration of mechanical ventilation. Although not the primary focus of this analysis, the relative abundances of dominant bacterial phyla (Actinobacteria, Bacteroidetes, Firmicutes, Fusobacteria, and Proteobacteria) were also examined. They showed no significant differences between groups (data not shown). A detailed analysis of compositional shifts is presented separately.

### Beta diversity of lower respiratory tract microbiota

**Fig. 3:**
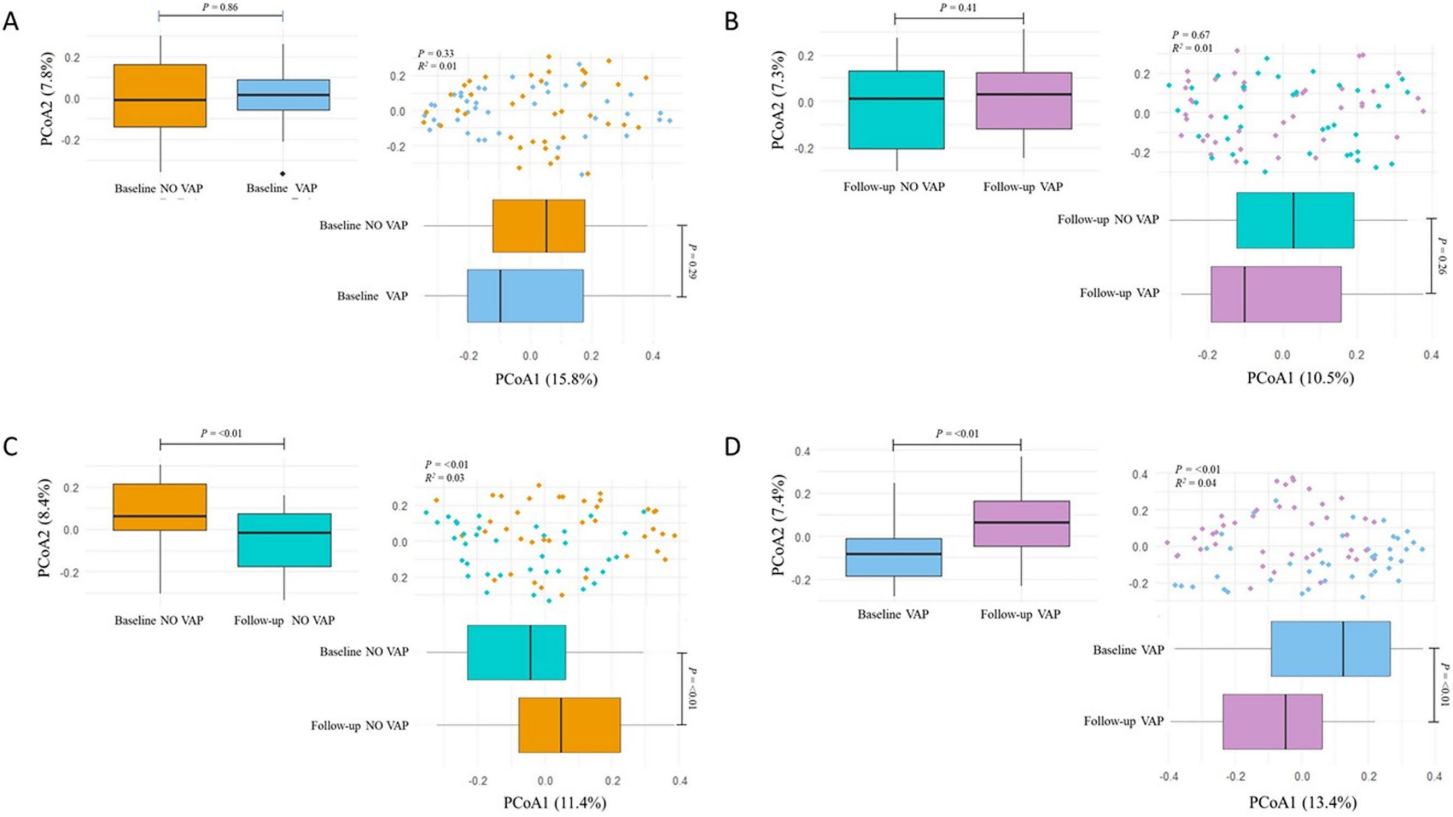
Beta Diversity Comparisons. Principal Coordinates Analysis (PCoA) plots illustrating beta diversity based on Bray-Curtis distances in baseline and follow-up samples from VAP and non-VAP patients. (**A**) Baseline samples show no significant difference in beta diversity between VAP and non-VAP groups. (**B**) Follow-up samples also show no significant difference. (**C**) Non-VAP patients exhibit significant changes in beta diversity between baseline and follow-up samples. (**D**) VAP patients similarly show significant differences between baseline and follow-up samples. Asterisks denote the level of significance observed: * = *p* ≤ 0.05; ** = *p* ≤ 0.01; *** = *p* ≤ 0.001; ns = ≥ 0.05

Beta diversity was analysed using Bray-Curtis distances to compare baseline and follow-up samples between VAP and non-VAP patients. PERMANOVA showed no significant differences in community composition at baseline (R² = 0.01, *p* = 0.33) or follow-up (R² = 0.01, *p* = 0.24) (Fig. 3A and B). The Kruskal-Wallis test confirmed these findings (baseline: PCoA1, *p* = 0.86; PCoA2, *p* = 0.33; follow-up: PCoA1, *p* = 0.41; PCoA2, *p* = 0.67), indicating that beta diversity remained stable between groups despite mechanical ventilation. No significant differences were detected at the community level; however, smaller shifts may not have been captured with the current analyses.

Within-group comparisons of baseline and follow-up samples revealed significant differences in beta diversity. In non-VAP patients, PCoA analysis showed R² = 0.03, *p* = 0.002, with Kruskal-Wallis results of *p* < 0.01 for PCoA1 and PCoA2 (Fig. 3C). Similarly, in VAP patients, PCoA analysis yielded R² = 0.04, *p* = 0.001, with Kruskal-Wallis results of *p* = 0.01 for PCoA1 and *p* < 0.01 for PCoA2 (Fig. 3D). Significant changes in BAL microbiota composition were observed over time in both groups, indicating temporal shifts associated with mechanical ventilation, regardless of VAP status.

### Cultures and microbial signatures differentiate VAP and non-VAP patients

Follow-up BAL cultures were negative in all non-VAP patients, supporting the clinical diagnosis and the absence of microbiologically confirmed lower respiratory tract infection. In contrast, patients who developed VAP had positive BAL cultures, with *Staphylococcus aureus*, *Klebsiella* spp., *Enterobacter cloacae*, and *Haemophilus influenzae* identified as the most frequent pathogens, occasionally appearing in coinfections (Fig. S4). To explore microbial community differences beyond cultivable pathogens, we conducted Random Forest analysis on genus-level taxonomic profiles from 16 S rRNA sequencing. This revealed that *Staphylococcaceae*, *Lachnospiraceae*, and *Methylobacteriaceae* were more abundant in patients with VAP, while *Streptococcaceae* and *Flavobacteriaceae* were more prevalent or stable in non-VAP patients (Fig. S5). Differences in microbial composition between VAP and non-VAP patients were observed even in the absence of culture-detectable pathogens, based on 16 S rRNA sequencing.

### Lower respiratory tract inflammation analysis

**Fig. 4:**
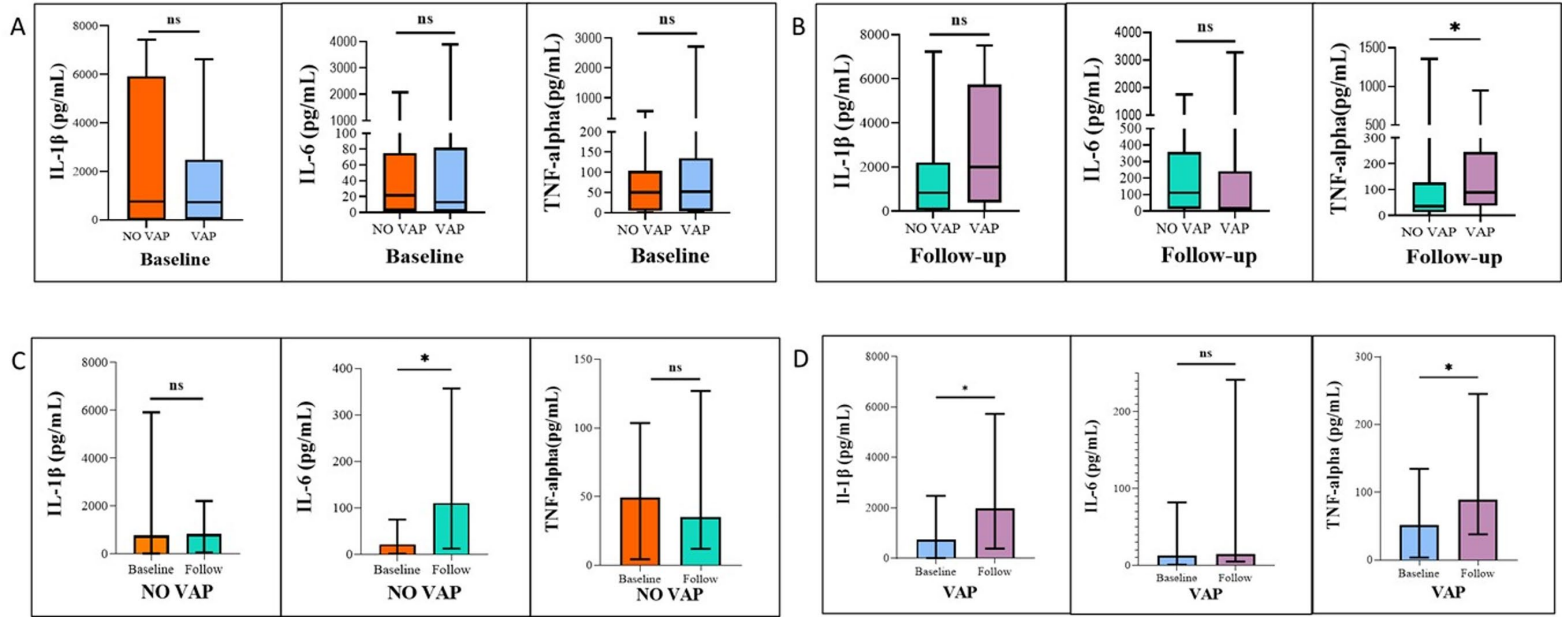
Temporal and Type-Specific Cytokine Variability in NO VAP and VAP Patients. BAL sample analysis illustrating cytokine levels (IL-1β, IL-6, TNF-α) at baseline and follow-up in VAP and non-VAP patients. (**A**) Baseline samples show no significant differences in cytokine levels between VAP and non-VAP groups. (**B**) Follow-up samples also show no significant differences, except for a significant increase in TNF-α levels in VAP patients compared to non-VAP patients. (**C**) Non-VAP patients exhibit no significant changes in cytokine levels between baseline and follow-up samples. (**D**) VAP patients show significant increases in IL-1β levels from baseline to follow-up, while TNF-α levels also increase significantly. Asterisks denote the level of significance observed: * = *p* ≤ 0.05; ** = *p* ≤ 0.01; *** = *p* ≤ 0.001; ns = ≥ 0.05

IL-1β, IL-6, and TNF-α concentrations in BAL were measured at admission and during follow-up. At admission, there were no significant differences between non-VAP and VAP patients for Il-1β (738.2 pg/mL [5.5–2477] vs. 764.0 pg/mL [4.9–5912]; *p* = 0.69), IL-6 (12.7 pg/mL [0.84–82.0] vs. 21.7 pg/mL [1.8–75.1]; *p* = 0.78), or TNF-α (51.9 pg/mL [3.5–134.6] vs. 49.6 pg/mL [4.3–103.7]; *p* = 0.83) (Fig. 4A). During follow-up, TNF-α levels significantly increased in VAP patients compared to non-VAP patients (88.6 pg/mL [37.9–245.3] vs. 35.1 pg/mL [11.9–127.1]; *p* = 0.02), while IL-1β and IL-6 remained unchanged (Fig. 4B).

Within the non-VAP group, IL-6 concentrations increased from baseline to follow-up (21.7 pg/mL [1.8–75.1] vs. 110.4 pg/mL [12.6–357.1]; *p* = 0.04), whereas IL-1β (764.0 pg/mL [4.9–5912] vs. 827.6 pg/mL [41.1–2200]; *p* = 0.97) and TNF-α (49.6 pg/mL [4.3–103.7] vs. 35.1 pg/mL [11.9–127.1]; *p* = 0.66) remained stable (Fig. 4C). In contrast, VAP patients exhibited a significant increase in IL-1β (738.2 pg/mL [5.5–2477] vs. 1987 pg/mL [386.5–5732]; *p* = 0.02) and TNF-α (51.9 pg/mL [3.5–134.6] vs. 88.6 pg/mL [37.9–245.3]; *p* = 0.04) from admission to follow-up, while IL-6 levels remained unchanged (12.7 pg/mL [0.84–82.0] vs. 14.6 pg/mL [4.8–241.6]; *p* = 0.23) (Fig. 4D). Taken together, these results show that IL-1β and TNF-α levels increased in patients who developed VAP, whereas IL-6 elevations were detected across all patients undergoing invasive mechanical ventilation (Table S3).

### Differences in lower respiratory tract metabolites between Non-VAP and VAP patients

The reliability of metabolomic data obtained via GC-QTOF-MS was validated using Principal Component Analysis. The Quality Control (QC) sample clustering within the PCA confidence ellipse (Fig. S6) confirmed the stability and reproducibility of the data. However, OPLS-DA revealed a low predictive capacity (Q2 ≤ 0.0204), indicating minimal metabolic differences between VAP and non-VAP patients at baseline and follow-up.

K-fold cross-validation was employed to assess the model’s predictive capability. Initial cross-validation analysis of variance (CV-ANOVA) values greater than 0.05 suggested potential overfitting, meaning the models might not generalise well to new data. However, the comparison between baseline and follow-up in the VAP group showed acceptable predictive performance (Q2 = 0.374), indicating moderate reliability in distinguishing these time points. Further K-fold validation yielded CV-ANOVA values < 0.05 (Fig. S7A). A permutation plot confirmed model validity, as permutation-derived Q2 values were lower than the original, and the regression line intersected the negative y-axis (Fig. S7B). All models demonstrated low predictive performance, with no evidence of overfitting based on validation metrics.

We identified 14 metabolites through univariate and multivariate analyses that met our criteria (*p* < 0.05 or Variable Importance in Projection [VIP] > 1). Most of these metabolites were increased in patients with VAP during follow-up compared to baseline values, as detailed in Table S4. Pathway enrichment analysis revealed that the altered metabolites were involved in several key metabolic pathways, including the pentose phosphate pathway, citric acid cycle, alanine, aspartate and glutamate metabolism, phenylalanine, tyrosine and tryptophan biosynthesis, pyruvate metabolism, glycolipid metabolism, and glycolysis/gluconeogenesis (Fig. S8).

## Discussion

This study analysed lung microbiome composition, inflammation, and metabolism in VAP patients under IMV. Alpha and beta diversity did not differ between groups, but longitudinal shifts were observed. In VAP, follow-up cultures identified *Staphylococcus aureus*,* Klebsiella pneumoniae*, and *Haemophilus influenzae*. Notably, *Pseudomonas aeruginosa*, a frequent VAP pathogen in other ICU populations, was not detected in our cohort. This may reflect the trauma-dominant profile of our patients, the absence of chronic lung disease, and local microbiological patterns [25, 26]. Despite separate analyses, these findings underscore key contributors to VAP pathogenesis and highlight the need for integrative studies to elucidate their interplay.

In this context, the relatively high incidence of VAP in our cohort (51%) may be explained by the clinical profile of the study population, which consisted predominantly of patients with traumatic brain injury and other neurological conditions. These groups are known to have an elevated risk of aspiration and VAP due to impaired consciousness and the need for invasive neuromonitoring. Previous studies have reported VAP rates between 20% and 42% in similar populations [27–30]. Additionally, all patients were enrolled in a structured surveillance protocol that included systematic BAL sampling and close clinical monitoring. This design may have increased VAP detection sensitivity compared to routine ICU settings, introducing a potential detection bias that could partially account for the high observed incidence.

Several studies have investigated the dynamics of the lung microbiome in mechanically ventilated patients and their relationship to ventilator-associated pneumonia (VAP). Zakharkina et al.. and Kelly et al.. reported reduced alpha diversity in VAP patients, suggesting a role for dysbiosis in its development [14, 31]. However, Emonet et al.. argued that these changes may result from clinical interventions (e.g., antibiotics, ventilation duration) rather than VAP [32]. In contrast, we found no significant differences in alpha diversity between VAP and non-VAP groups at baseline or follow-up. Unlike previous studies, our longitudinal analysis captured the temporal dynamics of microbes, offering a more comprehensive perspective. The homogeneity of our cohort, characterised by the absence of preexisting lung disease, lung infections, or prior antibiotic use, may have reduced variability. Moreover, VAP diagnosis remains challenging, as Clinical Pulmonary Infection Score (CPIS) has limited sensitivity, specificity, and interobserver agreement [33]. These findings suggest that alpha diversity alone cannot predict VAP, highlighting its multifactorial nature.

On the other hand, we observed significant beta diversity shifts in both patient groups, reflecting dynamic lung microbiome changes during mechanical ventilation. This suggests that mechanical ventilation, regardless of VAP development, influences microbial communities over time. Kitsios et al.., Kelly et al.., and Mouranni et al.. reported similar beta diversity shifts [31, 34, 35]. Such disruption may promote dysbiosis by shifting the balance toward potential pathogens. Consistently, Dickson et al.. demonstrated that hyperoxia, common in mechanically ventilated patients, alters lung microbial communities by favouring oxygen-tolerant taxa [36]. This highlights the difficulty in distinguishing microbiome changes induced by VAP from those driven by therapeutic interventions, such as oxygen therapy. These findings underscore the multifactorial nature of pulmonary microbiome alterations in critical illness, complicating efforts to identify disease-specific microbial signatures.

Our analysis reveals a progressive inflammatory response in patients with VAP. While IL-1β, IL-6, and TNF-α levels were similar at admission, IL-1β and TNF-α significantly increased during follow-up, indicating immune activation in later infection stages. IL-1β elevation aligns with Conway et al.., identifying it as a key pulmonary inflammation marker [37]. Similarly, TNF-α elevation supports Millo et al..’s findings of increased lung TNF-α in VAP [42], potentially reflecting immune dysregulation linked to microbiome alterations [45]. IL-6 levels remained unchanged in VAP but increased across the cohort, possibly reflecting an IMV-induced inflammatory response, complicating differentiation from VAP-related inflammation [38]. TNF-α, associated with necroptosis and pyroptosis, raises questions about whether its rise results from alveolar cell death or infection, requiring further investigation. These findings underscore the importance of integrating molecular and microbiological diagnostics with clinical criteria to more effectively stratify patients and characterise inflammation and microbiome dynamics in VAP [39, 40].

Metabolic shifts in the pentose phosphate pathway and citric acid cycle suggest a transition from aerobic to anaerobic metabolism in VAP, marked by increased pyruvic acid and decreased citric acid levels. These changes may reflect both host cellular adaptation to oxidative stress, potentially involving Nuclear factor erythroid 2-related factor 2 (Nrf2) activation and modulation of the NOD-like receptor, leucine-rich receptor, and pyrin domain-containing protein 3 (NLRP3) inflammasome, and microbial metabolic activity. Although the exact origin of the metabolites (host vs. microbial) cannot be determined in this study, the affected pathways are consistent with hypoxia-driven immune activation and tissue stress. This shift toward anaerobic glycolysis may serve as a compensatory mechanism while influencing immune signalling [41]. At follow-up, elevated levels of alanine, leucine, and tyrosine may indicate metabolic adaptation and tissue repair [42, 43], while increased urea could reflect altered disruptions of nitrogen metabolism or alveolocapillary permeability. These metabolic changes are consistent with prior findings showing that hypoxia and local inflammation favour the proliferation of anaerobic bacteria, particularly *Prevotella* and *Fusobacterium* spp., which may further amplify inflammation [44, 45]. Identifying early shifts in both metabolic and microbial profiles may enhance the assessment of VAP risk and inform targeted therapeutic strategies.

This study presents notable strengths and limitations. Although the sample size was modest and antibiotic exposure was not systematically controlled, the longitudinal design enabled the characterisation of dynamic changes in the microbiome, inflammatory response, and metabolome during mechanical ventilation. While detailed tracking of cumulative antibiotic days and class-specific exposures was not available, all VAP patients received antibiotics for ≥ 48 h before diagnosis, which may have influenced microbial and host response patterns. The studied population was relatively homogeneous, focusing on patients with non-infectious respiratory failure and minimal comorbidities, which may limit the generalizability of the findings to broader ICU populations. Additionally, the COVID-19 pandemic contributed to slower patient enrolment and limited the size of the eligible cohort. The use of 16 S rRNA sequencing, although it restricts taxonomic resolution and excludes viral and fungal communities, provides robust insights into bacterial community dynamics. In addition, total and differential BAL cell counts were not performed, as sample handling prioritised immediate preservation for omics-based analyses. The observed elevations in IL-1β and TNF-α highlight key inflammatory pathways associated with VAP, offering a foundation for future research in more diverse and heterogeneous patient populations.

**Fig. 5:**
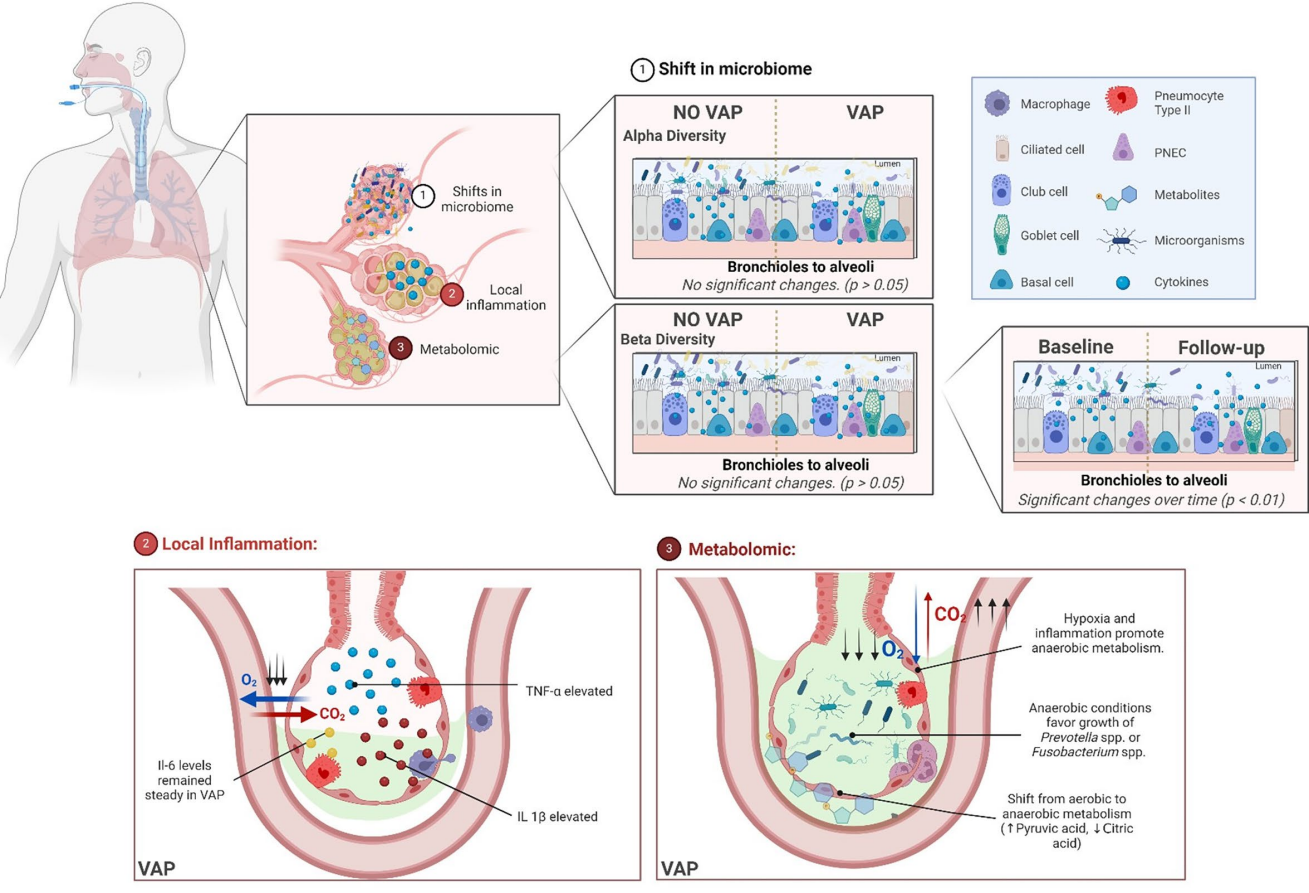
Lung Microbiome, Immune Response, and Metabolomic Dysregulation in Ventilator-Associated Pneumonia. This figure provides a comprehensive visual model of the lung microbiome, immune response, and metabolic dysregulation in ventilator-associated pneumonia (VAP)

In conclusion, the use of 16 S rRNA sequencing, although it restricts taxonomic resolution and excludes viral and fungal communities, provides robust insights into bacterial community dynamics. In this study, we comprehensively analysed the microbial, immune, and metabolic dynamics in the progression of VAP under IMV. We found significant shifts in beta diversity over time in both VAP and non-VAP patients, indicating that mechanical ventilation affects the BAL microbiota regardless of VAP status. IL-1β and TNF-α emerged as key immune markers, while disruptions in the pentose phosphate and citric acid cycles offer potential targets for early diagnosis and intervention. Further studies are needed to investigate taxonomy-specific changes and their relationships with VAP-related microorganisms. Larger cohorts are crucial to validate these findings, which could inform innovative strategies to reduce VAP severity and improve outcomes (Fig. 5).

## Electronic supplementary material

Supplementary Material 1

## Data Availability

The dataset supporting this article’s results has been posted to the NIH Sequence Read Archive (accession number PRJNA1212098). https://www.ncbi.nlm.nih.gov/bioproject/PRJNA1212098/.
