# Supplementary material for: Longitudinal analysis of lung microbiome, immune response, and metabolism in ventilator-associated pneumonia: a cohort study
Source: Crit Care. 2025 Jul 3;29:275. doi: 10.1186/s13054-025-05498-1 (PMC12231915; doi:10.1186/s13054-025-05498-1)
Supplement: Supplementary file 1 — Supplementary Material 1 [file 13054_2025_5498_MOESM1_ESM.docx]

**TITLE**: Longitudinal Analysis of Lung Microbiome, Immune Response, and Metabolism in Ventilator-Associated Pneumonia: A Cohort Study.

**AUTHORS**: Ingrid G. Bustos^1,2^, Cristian C. Serrano-Mayorga^1,2,3^, José L. Guerrero^4^, Jennifer M. Baker^5,6,7^, Christopher Brown^8^, Nicole Falkowski^5^, Piyush Ranjan^5^, Alejandro Acosta-Gonzalez^1^, Lina M. Mendez^3^, Acenet Garcia-Cordoba^3^, Adriana Echeverry-Gutierrez^3^, Denis A. Bojaca^3^, Marcela Chisica-Mahecha^3^, Nicol Guarin-Tequia^3^, Lilina Romero-Romero^3^, Norberto Gonzalez-Juarbe^9^, Alejandro Rodriguez^10^, Mónica P. Cala^4^, Ignacio Martin-Loeches^11^, Sanjay H. Chotirmall^12,13^, Robert P. Dickson^5,6.7^ and Luis F. Reyes^1,3,14*.^

**Online Data Supplement:** This article includes an online data supplement, accessible in the electronic version of this issue at Intensive Care Medicine's website.

**DATA SUPPLEMENT**

**MATERIALS AND METHODS**

*Quantification of 16S rRNA gene copy number*

Bacterial DNA in Bronchoalveolar lavage (BAL) samples and negative controls were quantified using a QX200 Droplet Digital Polymerase Chain Reaction (ddPCR) (Bio-Rad, Hercules, CA). Primers and cycling conditions were performed according to a previously published protocol (75) using the Bio-Rad C1000 Touch Thermal Cycler. Specifically, primers were 5′-GCAGGCCTAACACATGCAAGTC-3′ (63F) and 5′-CTGCTGCCTCCCGTAGGAGT-3′ (355R) with initial denaturation at 95°C for 5 min, 40 cycles at 95°C for 15 s and 60°C for 1 min, 1 cycle at 4°C for 5 min, and 1 cycle at 90°C for 5 min, all at a ramp rate of 2°C/s. Droplets were subsequently quantified using the Bio-Rad Quant iSOFT software. Two replicates were used per sample.

*Sample preparation for metabolomics*

BAL samples underwent centrifugation at 16,000 rpm and 4°C for 10 minutes, and supernatant was collected for metabolomic analysis. For metabolite extraction of BAL samples, 100 µL of BAL fluid with 400 µL of cold methanol: chloroform (9:1) at -20°C for 5 minutes, followed by centrifugation at 14,000 rpm and 4°C for 15 minutes, following the procedure outlined by Cala *et al.(1)* . The resulting supernatant was utilized for gas chromatography quadrupole time-of-flight mass spectrometry (GC-QTOF-MS) analysis. Samples were then prepared for GC-QTOF-MS analysis by drying 20 µL of extracted plasma samples for 1.5 hours at 35°C, adding 10 µL of O-methoxyamine in pyridine (15 mg/mL), vortexing for 10 minutes, and incubating in darkness for 16 hours. Subsequently, 10 µL of N, O-bistrifluoroacetamide (BSTFA) with 1% trimethylchlorosilane (TMCS) were added and incubated at 70°C for 1 hour. Finally, 60 µL of methyl stearate in heptane (10 mg/L) was added as an internal standard, followed by vortexing for 10 minutes, in line with the methods outlined by (2-4). Quality control samples, created by combining equal volumes of all BAL samples, were injected into the system every 10 samples to ensure analytical stability.

*Metabolomics analysis by GC-QTOF-MS*

Metabolomics analysis was conducted using a gas chromatography–quadrupole time-of-flight mass spectrometer (GC-QTOF; Agilent Technologies 7890B GC coupled to a QTOF 7250, Agilent Technologies, Waldbronn, Germany). A 1 µL volume of the derivatized sample was chromatographically separated on an HP-5MS UI column (30 m x 0.25 mm x 0.25 µm), with helium as the carrier gas at a constant flow rate of 0.7 ml/min. The injector temperature was maintained at 280 °C, and a split ratio of 30:1 was employed. The temperature gradient program started at 60 °C for 1 minute, then ramped up to 325 °C at 10 °C/min. The gas chromatography–mass spectrometry (GC-MS) transfer line was set at 280 °C, the filament source at 250 °C, and the quadrupole at 150 °C. The electron ionization source operated at 70 eV, and the mass spectrometer functioned in full scan mode, ranging from 50 to 600 m/z at a scan rate of 5.00 scans/s.

*Metabolomics data processing and metabolite identification*

Data deconvolution and alignment were carried out for the GC-QTOF-MS data using the Agilent MassHunter Unknowns Analysis B.10.00 software and Agilent MassProfiler Professional, respectively. The processed data was subsequently exported to the Agilent MassHunter Quantitative software for integration, and the metabolite areas were normalized based on the response of the quality control samples using the freely available SERRF software (https://slfan.shinyapps.io/ShinySERRF/). Subsequently, the data underwent filtering for presence and reproducibility, retaining only those metabolites present in a minimum of 80% of the samples and exhibiting a coefficient of variation of less than 30% in the quality control samples.

The process of identifying metabolites derived through GC-QTOF-MS commenced with the deconvolution of total ion chromatograms, utilizing Agilent Unknowns Analysis B.10.0 software. This crucial step involved comparing the extracted metabolites' retention time and mass spectrum with those cataloged in the Fiehn GC-MS Metabolomics RTL (Retention Time Locked) Library (5). It is imperative to highlight that the degree of identification attained using this platform was categorized as level 1, corresponding to the highest confidence in metabolite annotation, signifying direct matching with authentic standards in the library. Based on the comparison, annotation levels were reported for each platform according to the Metabolomics Standards Initiative (6).

**FIGURES**

**Fig S1.** Aetiologies of ICU Admission with Mechanical Ventilation Requirement.

**
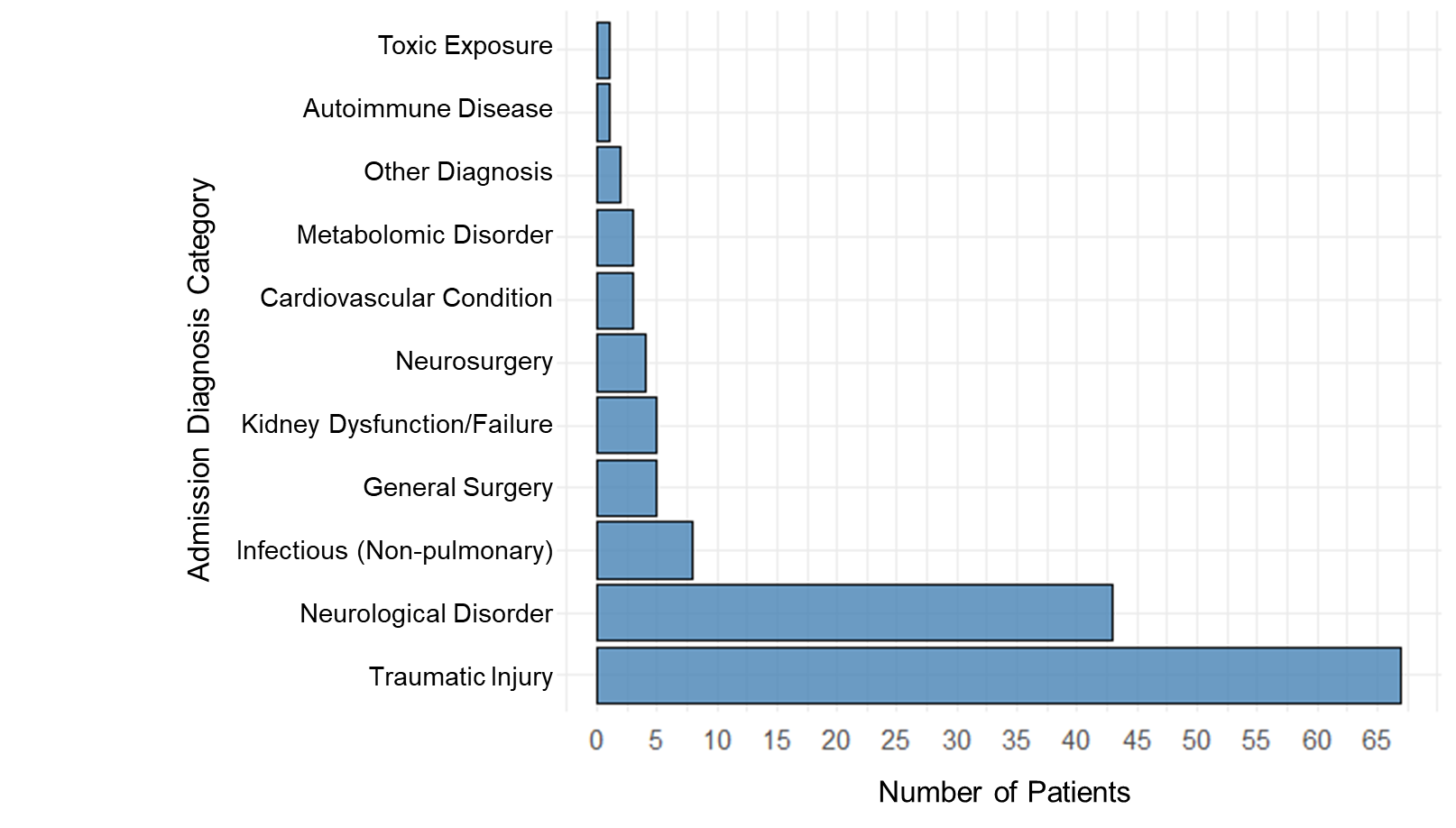
**

**Fig. S2. Rarefaction curves.** This rarefaction curve graphically compares the microbial diversity in BAL samples from patients with VAP, patients without VAP (NO VAP), and negative controls based on 16S rRNA gene sequencing. The graph plots the number of unique microbial species identified against the number of sequencing reads.


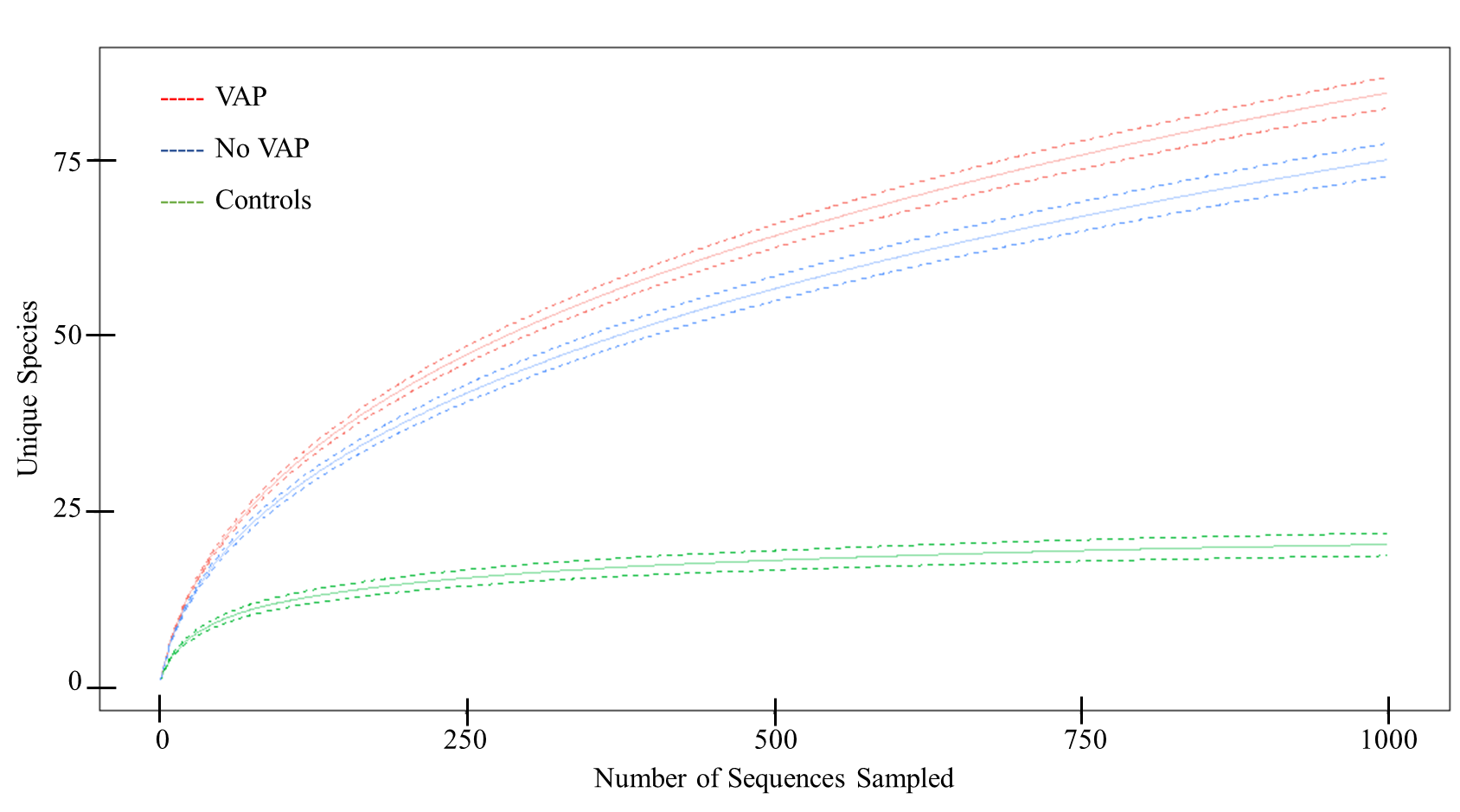


**Fig. S3. Absolute Abundance of DNA Copies.** The absolute abundance of DNA copies evaluated in control and BAL samples at baseline and follow-up time points in both VAP and non-VAP patients using droplet digital PCR (ddPCR).

**
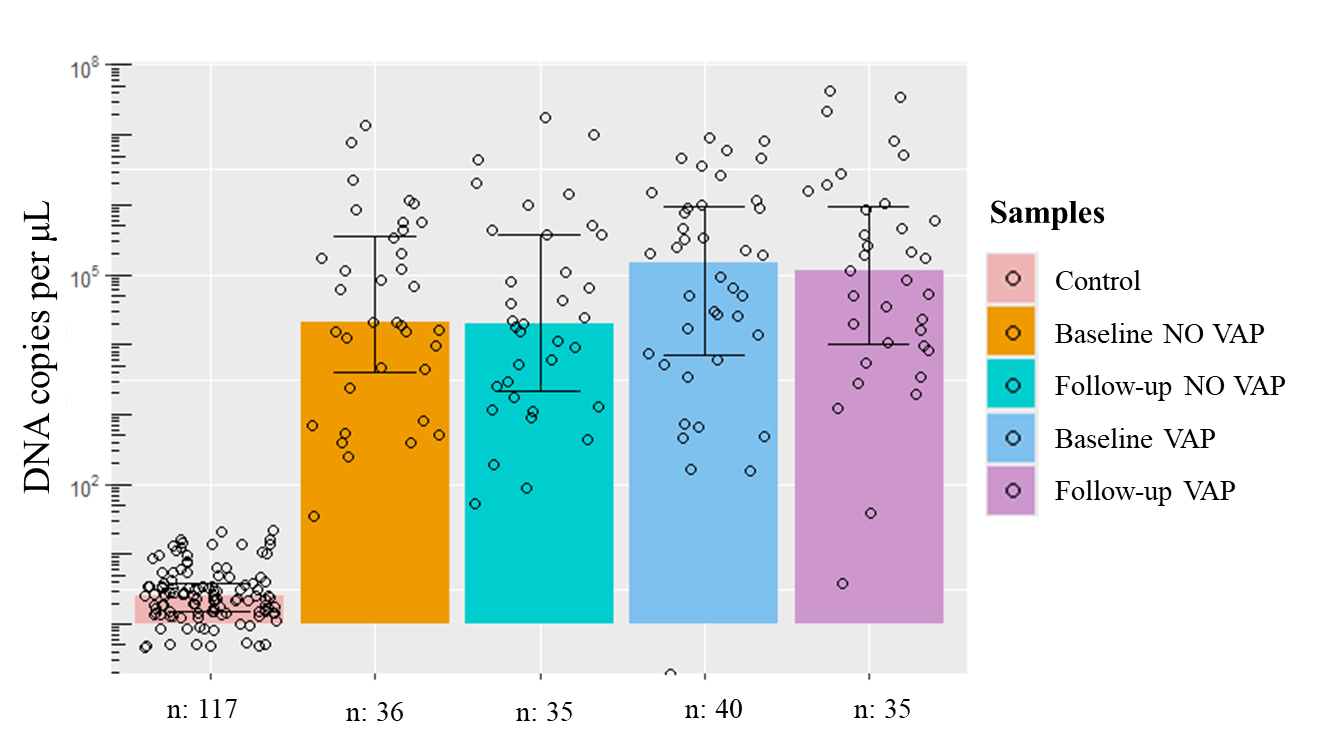
**

**Fig. S4. Microorganisms Detected Conventionally in VAP Patients.** The figure shows the frequency of isolated microorganisms and co-infections in VAP patients. The upper bar chart highlights the prevalence of single-pathogen infections, with a few cases of multiple-pathogen co-infections. The intersections below illustrate specific combinations of microorganisms found in co-infected patients.

**
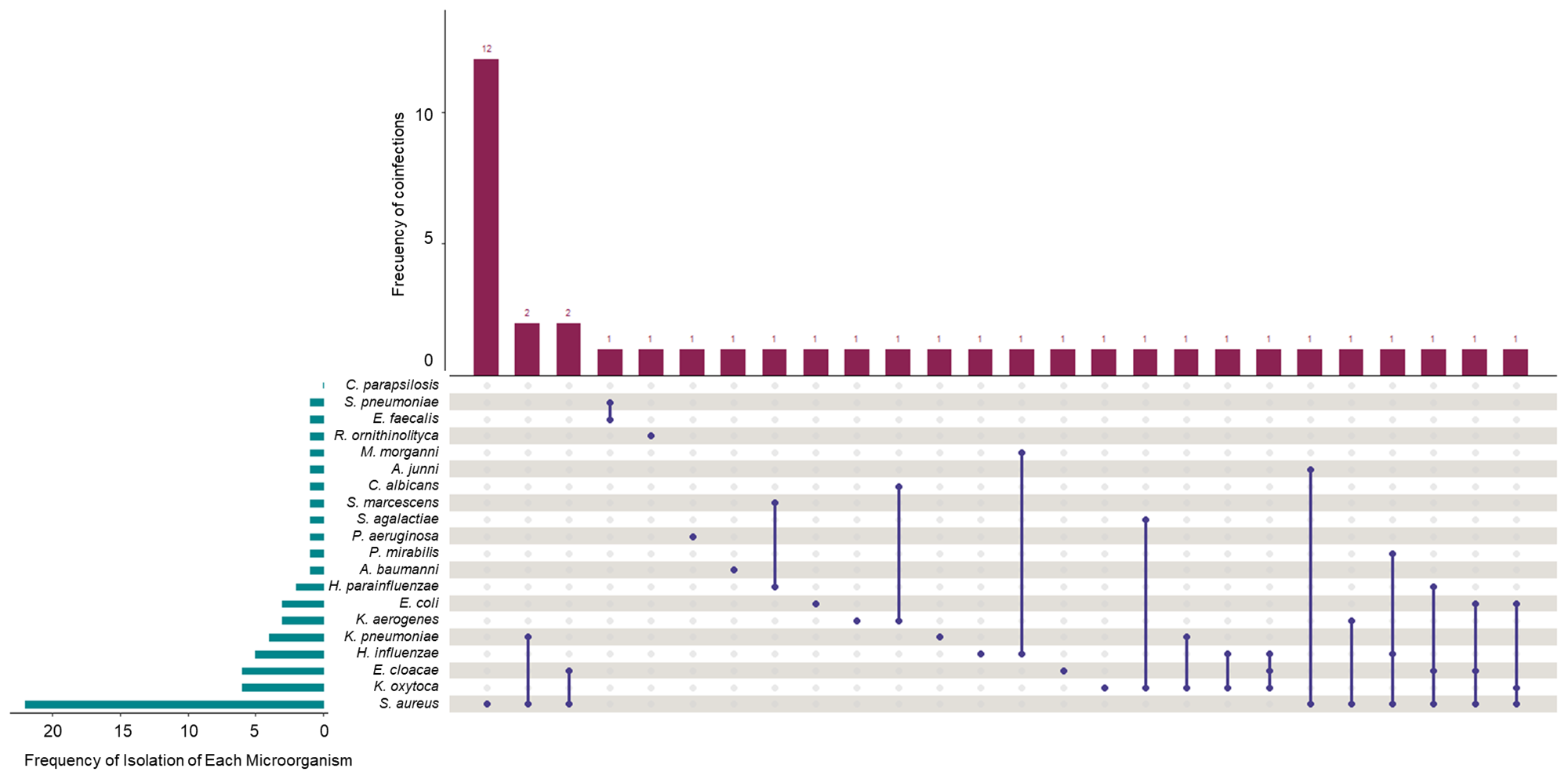
**

**Fig. S5. Importance of Microbial Families in Random Forest Classification:** The plots show the key microbial families contributing to the classification accuracy of the Random Forest model for non-VAP (Panel A) and VAP patients (Panel B). The "Mean Decrease in Accuracy" reflects the importance of each microbial family in model performance, where higher values indicate greater significance for classification accuracy.


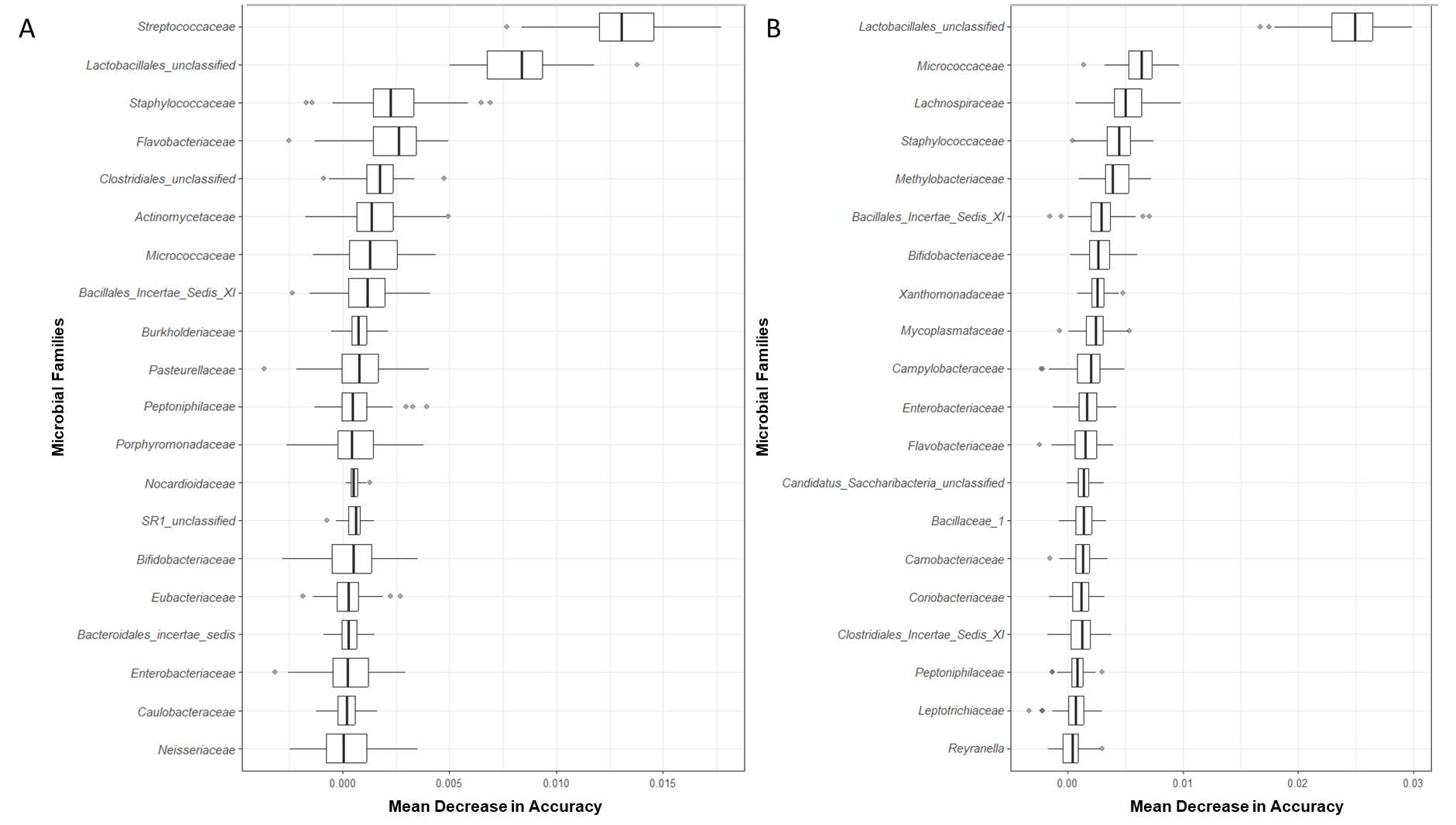


**Fig. S6.** **Analytical Quality Consistency in GC/MS-QTOF**: An OPLS-DA score plot highlighting the quality control consistency (orange point) within a multivariate feature space, demonstrating a robust cumulative model fit (R²X(cum): 0.825) and predictive capability (Q²(cum): 0.775).


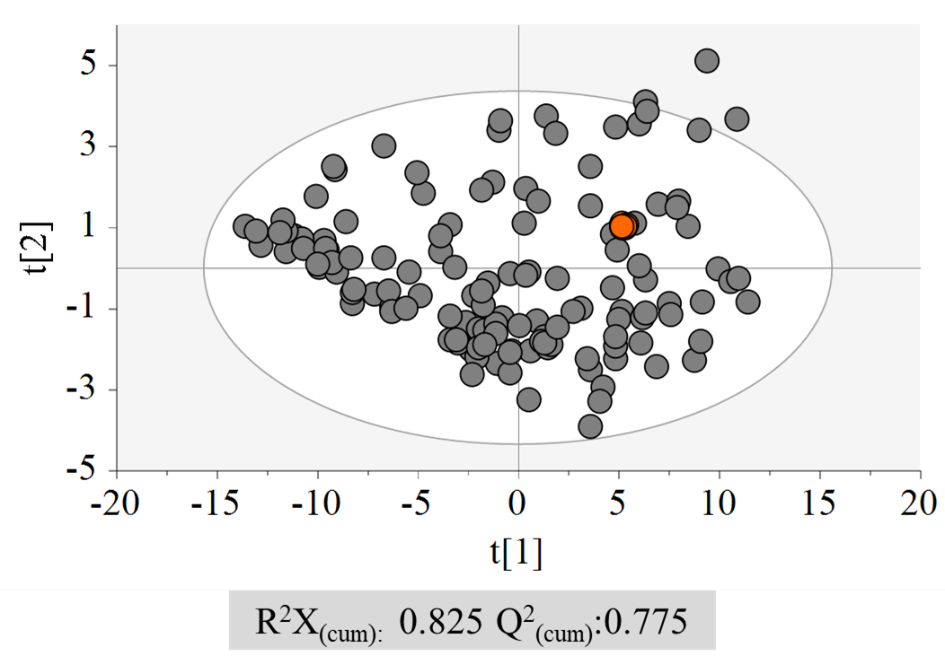


**Fig S7. OPLS-DA Score Plots.** **A**. (Green dots, Baseline VAP; blue dots, Follow-up VAP), Metabolomics by GC-QTOF-MS: R2(cum): 0.691, Q2 (cum): 0.374, *p _cv-anova_*: 1.47e-04, **B**. Permutation plot (n=200). green dots, R2; blue squares Q2.

**
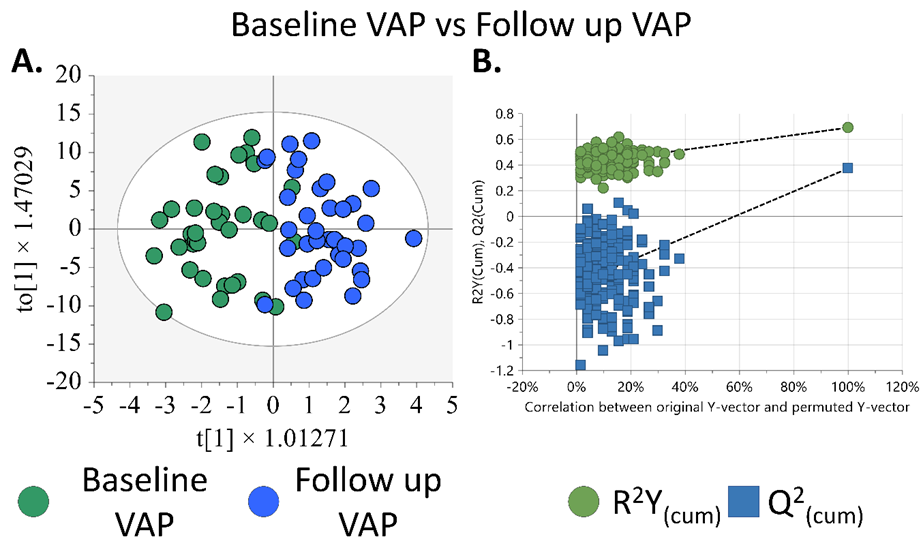
**

**Fig S8. Analysis of altered local metabolic pathways.** The y-axis shows statistical significance (–log₁₀ *p*), and the x-axis shows pathway impact. Circle size indicates the number of metabolites involved; color intensity (yellow to red) reflects the degree of change.

**
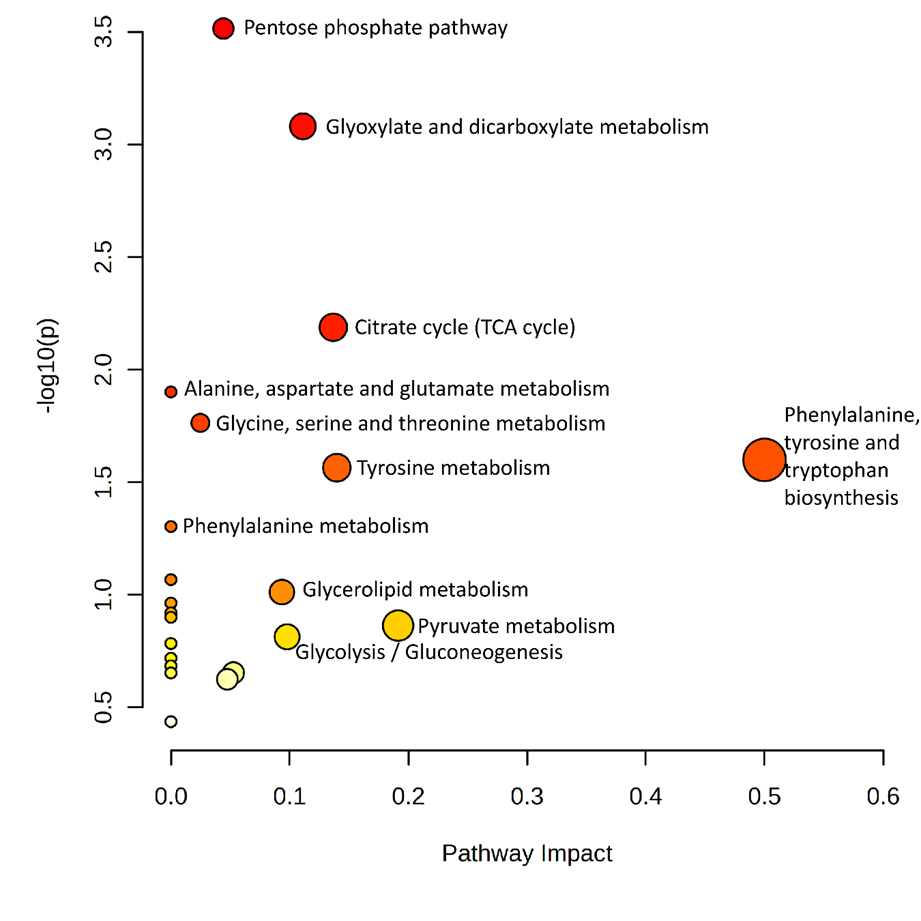
**

**TABLES**

**Table S1**. Demographic Data and Outcomes of Enrolled Patients.

| **Characteristic** | **All**  **n = 80** | **VAP**  **n = 41** | **No-VAP**  **n = 39** | **p-value** |
| --- | --- | --- | --- | --- |
| **Demographic** | | | | |
| Male. n (%) | 55 (68.8) | 23 (56.1) | 32 (82.1) | **0.02** |
| Age. median (IQR) | 51.5 (34.0-69.2) | 48.0 (34.0-67.0) | 53.0 (35.0-70.0) | 0.47 |
| **Comorbid conditions. n (%)** | | | | |
| Alcohol | 4 (5.0) | 1 (2.4) | 3 (7.7) | 0.57 |
| Stroke | 3 (3.8) | 1 (2.4) | 2 (5.1) | 0.96 |
| Cancer | 3 (3.8) | 2 (4.9) | 1 (2.6) | 1 |
| Diabetes mellitus | 7 (8.8) | 2 (4.9) | 5 (12.8) | 0.38 |
| Coronary disease | 2 (2.5) | 1 (2.4) | 1 (2.6) | 1 |
| Mental Illness | 1 (1.3) | 0 (0.0) | 1 (2.6) | 0.97 |
| Chronic kidney disease | 6 (7.5) | 2 (4.9) | 4 (10.3) | 0.62 |
| COPD | 3 (3.8) | 0 (0.0) | 3 (7.7) | 0.22 |
| Cardiac failure | 2 (2.5) | 1 (2.4) | 1 (2.6) | 1 |
| Hemodialysis | 2 (2.5) | 0 (0.0) | 2 (5.1) | 0.45 |
| Arterial hypertension | 27 (33.8) | 11 (26.8) | 16 (41.0) | 0.26 |
| Obesity | 5 (6.3) | 3 (7.3) | 2 (5.1) | 1 |
| Smoking | 6 (7.5) | 2 (4.9) | 4 (10.3) | 0.62 |
| No background | 40 (50.0) | 23 (56.1) | 17 (43.6) | 0.37 |
| **Outcomes** | | | | |
| Length of stay in ICU, days (IQR) | 13.0 (7.7-16.0) | 15.0 (11.0-24.0) | 10.0 (6.0-13.5) | **0.01** |
| Length of stay in the hospital, days (IQR) | 18.0 (11.0-38.2) | 29.0 (12.0-49.0) | 15.0 (10.5-22.5) | **<0.01** |
| Intubation time, days (IQR) | 8.0 (5.0-11.0) | 10.0 (7.0-15.0) | 6.0 (5.0-8.5) | **<0.01** |
| Hospital Mortality (%) | 23 (28.8) | 12 (29.3) | 11 (28.2) | 1 |
| Mortality 28d (%) | 26 (32.5) | 13 (31.7) | 13 (33.3) | 1 |
| Mortality 90d (%) | 28 (35.0) | 15 (36.6) | 13 (33.3) | 0.94 |

**Table S2**. Physiological, Laboratory, and Scoring Data at Admission and During Follow-Up of Enrolled Patients.

| **Characteristic** | **All**  **n = 80** | **VAP**  **Baseline**  **n = 41** | **No-VAP**  **Baseline**  **n = 39** | **p-value** | **VAP follow-up**  **n = 41** | **No VAP follow-up**  **n = 39** | **p-value** |
| --- | --- | --- | --- | --- | --- | --- | --- |
| **Physiological Variables at Admission and Subsequent Follow-Up: Median and Interquartile Range (IQR)** | | | | | | | |
| Heart rate. BPM | 80.0 (65.2-96.5) | 77.0 (70.0-92.0) | 88.0 (73.5-102.2) | 0.06 | 78.0 (69.0-89.0) | 78.0 (66.0-90.0) | 0.76 |
| Respiratory rate. RPM | 18.0 (16.0-20.0) | 18.5 (17.0-20.0) | 18.0 (16.0-20.0) | 0.59 | 20.0 (18.0-22.0) | 20.0(18.0-20.0) | 0.31 |
| Temperature. °C | 36.5 (36.2-36.9) | 36.6 (36.3-37.0) | 36.4 (36.2-36.8) | 0.23 | 37.0 (36.9-37.7) | 36.9 (36.5-37.0 ) | **0.03** |
| SBP. mmHg | 119.0 (104.8-133.0) | 123.0 (108.0-132.0) | 118.0 (101.0-138.5) | 0.90 | 125.0 (119.0-138.0) | 129.0 (120.0-146.0) | 0.37 |
| DBP. mmHg | 69.0 (57.0-79.0) | 68.0 (57.0-76.0) | 70.5 (58.0-84.5) | 0.29 | 68.0 (62.0-74.0 ) | 68.0 (58.0-74.0) | 0.87 |
| PAM. mmHg | 10.2.1 (87.5-115.0) | 103.3 (88.3-111.0) | 101.3 (85.2-123.5) | 0.72 | 105.6 (99.6-118.0) | 108.3 (100.0-122.3) | 0.46 |
| SPO2. (%) | 93.0 (91.0-95.0) | 92.0 (92.0-96.0) | 93.0 (91.0-94.0) | 0.59 | 93.0 (91.0-94.0) | 94.0 (92.0-96.0) | 0.09 |
| Glasgow | 7.0 (6.0-10.0) | 7.0 (6.0-8.0) | 8.0 (6.0-13.0) | **0.04** | 7.0 (6.0-9.0) | 7.0 (6.0-10.0) | 0.27 |
| **Laboratory variables at admission and Subsequent Follow-up: Median and Interquartile Range (IQR)** | | | | | | | |
| WBC, cell x 103 | 13.1 (9.7-16.0) | 13.3 (11.1-15.9) | 11.8 (9.0-16.2) | 0.34 | 10.0 (8.5-13.5) | 9.95 (7.85-13.0) | 0.44 |
| Neutrophiles, (%) | 81.0 (70.7-87.0) | 81.0 (71.0-87.0) | 81.0 (71.7-87.2) | 0.86 | 79.5 (75.0-84.0) | 78.0 (76.0-83.0) | 0.82 |
| Hemoglobin, g/dL | 13.0 (11.6-14.0) | 13.3 (11.8-14.5) | 12.5 (11.3-13.4) | 0.09 | 9.7(8.5-11.1) | 9.7 (8.6-10.9) | 0.99 |
| Platelet, cell x 103 | 195.0 (157.2-270.0) | 190.0 (160.0-270.0) | 205.0 (160.0-270.0) | 0.95 | 165.0 (127.5-190.0) | 130.0 (120.0-172.5) | **0.04** |
| Creatinine, mg/dL | 1.0 (0.8-1.3) | 1.0 (0.8-1.3) | 0.9 (0.7-1.2) | 0.31 | 0.8 (0.6-1.1) | 0.8 (0.7-1.4) | 0.33 |
| BUN, mg/dL | 16.0 (12.0-21.5) | 15.0 (12.0-19.2) | 17.5 (13.7-26.0) | 0.11 | 19.0 (12.0-32.0) | 18.0 (12.0-27.2) | 0.63 |
| Blood glucose, mg/dL | 130.0 (120.0-160.0) | 130.0 (120.0-160.0) | 140.0 (120.0-165.0) | 0.83 | 140.0 (130.0-165.0) | 130.0 (117.5-150.0) | **0.03** |
| Sodium, mEq/L | 140.0 (138.0-142.0 ) | 140.0 (138.0-142.2) | 140.0 (138.0-141.5) | 0.86 | 143.0 (139.0-145.0) | 141.0 (139.0-147.5) | 0.62 |
| Potassium, mEq/L | 4.0 (3.6-4.5) | 4.0 (3.5-4.5) | 4.0 (3.7-4.5) | 0.54 | 3.8 (3.6-4.2) | 4.0 (3.7-4.4) | 0.22 |
| pH | 7.3 (7.2-7.4) | 7.3 (7.2-7.3) | 7.3 (7.2-7.4) | 0.17 | 7.4 (7.4-7.4) | 7.4 (7.4-7.4) | 0.76 |
| PCO2, mmHg | 36.0 (33.0-42.0) | 36.0 (33.0-39.2) | 37.0 (33.0-44.0) | 0.27 | 35.5 (32.0-40.0) | 37.5 (32.0-42.0) | 0.46 |
| PaO2, mmHg | 78.0(69.7-94.0) | 76.0 (69.0-88.0) | 84.0 (74.0-108.0) | 0.07 | 75.0 (71.0-81.7) | 81.5 (70.5-90.7) | 0.21 |
| FiO2 | 35.0 (28.0-41.2) | 32.0 (28.0-36.1) | 35.0 (28.0-45.0) | 0.71 | 30.0 (28.0-32.0) | 31.0 (28.0-35.0) | 0.14 |
| HCO3, mmol/L | 20.0 (17.0-23.0) | 20.0 (18.0-23.0) | 20.5 (16.7-23.0) | 0.73 | 25.0 (21.2-26.0) | 24.0(22.0-26.2) | 0.87 |
| Acid lactic, mmol/L | 1.9 (1.2-3.2) | 1.5 (0.9-3.2) | 2.2 /(1.4-3.1) | 0.17 | 1.1 (0.9-1.4) | 1.1 (0.9-1.3) | 0.62 |
| PT, seconds | 11.0 (10.0-13.0) | 11.0 (10.0-12.0) | 12.0 (10.5-13.5) | 0.14 | 11.0 (10.0-12.5) | 15.0 (12.5-15.7) | **0.05** |
| PTT, seconds | 24.0 (23.0-27.0) | 24.0 (22.0-26.0) | 25.0 (23.0-28.0) | 0.42 | 29.0 (28.0 -34.0) | 26.0 (26.0-27.0) | **0.01** |
| **Scores at admission and Subsequent Follow-up: Median and Interquartile Range (IQR)** | | | | | | | |
| SOFA | 8.0 (7.0-9.0) | 8.0 (6.0-9.0) | 8.0 (7.0-9.0) | 0.18 | 9.0 (7.0-10.0) | 9.0 (7.0-10.0) | 0.82 |
| APACHE | 16.0 (10.5-19.5) | 15.5 (9.0-19.2) | 17.0(13.0-21.7) | 0.16 | 14.0 (11.0-20.0) | 17.0 (14.0-19.0 ) | 0.15 |
| CPIS | 2.0 (1.0-3.0) | 2.0 (1.0-4.2) | 2.0 (1.0-3.0) | 0.54 | 3.0 (1.75-4.0) | 2.0 (1.0-2.0) | **<0.01** |

**Table S3.** Cytokine values.

| **Group** | **Cytokine** | **Baseline** | **Follow-up** | **p-value** |
| --- | --- | --- | --- | --- |
| Patients without VAP | Il-1β | 764.0 (4.9-5912.0) | 827.6 (41.1-2200.0) | 0.97 |
|  | Il-6 | 21.77 (1.8-75.1) | 110.4 (12.6-357.1) | 0.06 |
|  | TNF-alpha | 49.6 (4.3-1.3-7) | 35.1 (11.9-127.1) | 0.09 |
| Patients with VAP | Il-1β | 738.2 (5.5-2477.0) | 1987 (386.5-5732.0) | **0.02** |
|  | Il-6 | 12.77 (0.8-82.0) | 14.6 (4-241.6) | 0.19 |
|  | TNF-alpha | 51.9 (3.5-134.6) | 88.6 (37.9-245.3) | 0.08 |

Abbreviation: Il: Interleukin. TNF: tumor necrosis factor. VAP: ventilator-associated pneumonia.

**Table S4**. Identifying Significant Metabolites in Intubated Patients: A Comparative Study of Baseline and Follow-up Levels.

|  |  |  |  |  |  | **Baseline VAP vs Follow up VAP** | | |
| --- | --- | --- | --- | --- | --- | --- | --- | --- |
| **Compound** | **Formula** | **Target ion** | **RT (min)** | **^a^CV for QC (%)** | **Identification level** | **^b^Fold Change** | **^c^VIP** | ***^d^p* value** |
| ***Amino acids, peptides, and analogues*** |  |  |  |  |  |  |  |  |
| Alanylleucine | C_9_H_18_N_2_O_3_ | 202.13 | 15.39 | 9.71 | 2 | 0.67 | 1.10 | - |
| Tyrosine | C₉H₁₁NO₃ | 181.07 | 17.89 | 4.55 | 2 | 0.77 | 1.14 | - |
| ***Benzene and substituted derivatives*** |  |  |  |  | - |  |  |  |
| Phenyllactic acid | C₉H₁₀O₃ | 166.06 | 14.05 | 4.68 | 2 | 0.78 | 1.13 | **0.028** |
| ***Carboxylic acids and derivatives*** |  |  |  |  |  |  |  |  |
| Citric acid | C₆H₈O₇ | 192.03 | 16.73 | 12.79 | 2 | 0.32 | 1.22 | **0.005** |
| Pyruvic acid | C₃H₄O₃ | 88.02 | 6.70 | 2.49 | 2 | 1.24 | - | **0.008** |
| ***Diazines*** |  |  |  |  |  |  |  |  |
| Uracil | C₄H₄N₂O₂ | 112.03 | 10.90 | 2.94 | 2 | 1.07 | 1.15 | - |
| ***Fatty Acyls*** |  |  |  |  |  |  |  |  |
| Methylglutamic acid | C₆H₁₁NO₄ | 144.04 | 13.60 | 9.68 | 2 | 0.35 | 1.21 | **0.043** |
| ***Hydroxy acids and derivatives*** |  |  |  |  |  |  |  |  |
| Glycolic acid | C₂H₄O₃ | 76.02 | 7.08 | 2.93 | 2 | 0.72 | - | **0.015** |
| ***Organic carbonic acids and derivatives*** |  |  |  |  |  |  |  |  |
| Urea | CH₄N₂O | 60.03 | 9.41 | 3.41 | 2 | 0.09 | 2.50 | **<0.001** |
| ***Carbohydrates and carbohydrate conjugates*** |  |  |  |  |  |  |  |  |
| Gluconic acid | C₆H₁₂O₇ | 196.06 | 18.07 | 4.46 | 2 | 0.46 | 1.71 | **0.031** |
| Acetyl-mannosamine | C₈H₁₅NO₆ | 221.09 | 18.90 | 4.94 | 2 | 0.78 | 1.44 | **0.010** |
| Gluconic acid lactone | C₆H₁₀O₆ | 178.05 | 17.09 | 10.06 | 2 | 0.90 | 1.09 | - |
| Digitoxose | C₆H₁₂O₄ | 148.07 | 13.96 | 3.43 | 2 | 1.17 | 1.13 | - |
| Glyceric acid | C₃H₆O₄ | 106.03 | 10.83 | 3.54 | 2 | 0.81 | - | **0.016** |
| ^a^CV, coefficient of variation in the metabolites in the QC samples; ^b^Change, fold change in the abundance of the specified comparison calculated as (case/control), where the sign indicates the direction of change in the case group; ^c^VIP, variable importance in projection; ^d^p value * corresponding to the p values calculated by the Benjamini-Hochberg false discovery rate post hoc correction (FDR < 0.05). GC: gas chromatography, QTOF-MS: quadrupole time-of-flight mass spectrometer. | | | | | | | | |

**REFERENCES**

1. Cala MP, Aldana J, Medina J, Sanchez J, Guio J, Wist J, Meesters RJW. Multiplatform plasma metabolic and lipid fingerprinting of breast cancer: A pilot control-case study in Colombian Hispanic women. *PLoS One* 2018; 13: e0190958.

2. Garcia A, Barbas C. Gas chromatography-mass spectrometry (GC-MS)-based metabolomics. *Methods Mol Biol* 2011; 708: 191-204.

3. Cala MP, Meesters RJ. Comparative study on microsampling techniques in metabolic fingerprinting studies applying gas chromatography-MS analysis. *Bioanalysis* 2017; 9: 1329-1340.

4. Raczkowska BA, Mojsak P, Rojo D, Telejko B, Paczkowska-Abdulsalam M, Hryniewicka J, Zielinska-Maciulewska A, Szelachowska M, Gorska M, Barbas C, Kretowski A, Ciborowski M. Gas Chromatography-Mass Spectroscopy-Based Metabolomics Analysis Reveals Potential Biochemical Markers for Diagnosis of Gestational Diabetes Mellitus. *Front Pharmacol* 2021; 12: 770240.

5. Kind T, Wohlgemuth G, Lee DY, Lu Y, Palazoglu M, Shahbaz S, Fiehn O. FiehnLib: mass spectral and retention index libraries for metabolomics based on quadrupole and time-of-flight gas chromatography/mass spectrometry. *Anal Chem* 2009; 81: 10038-10048.

6. Blazenovic I, Kind T, Ji J, Fiehn O. Software Tools and Approaches for Compound Identification of LC-MS/MS Data in Metabolomics. *Metabolites* 2018; 8.
